# Magnitude and predictors of post-stroke cognitive impairment among Ethiopian stroke survivors: A facility-based cross-sectional study

**DOI:** 10.21203/rs.3.rs-2852302/v1

**Published:** 2023-05-18

**Authors:** Yared Zewde, Atalay Alem, Susanne K. Seeger

**Affiliations:** Addis Ababa University; Addis Ababa University; University of Wisconsin–Madison

**Keywords:** Cognitive impairment, Dementia, Stroke survivors, Predictors, Ethiopia

## Abstract

**Background:**

Stroke is emerging as a public health threat in sub-Saharan African countries including Ethiopia. Even though cognitive impairment is increasingly being recognized as a major cause of disability among stroke survivors there is a paucity of information on the magnitude of stroke-related cognitive dysfunction in Ethiopia. Thus, we assessed the magnitude and predictors of post-stroke cognitive impairment among Ethiopian stroke survivors.

**Methods:**

A facility-based cross-sectional study was employed to assess the magnitude and predictor of post-stroke cognitive impairment among adult stroke survivors who came for follow-up at least 3 months after the last stoke episode from February to June 2021 in three outpatient neurology clinics in Addis Ababa, Ethiopia. We used the Montreal Cognitive Assessment Scale-Basic (MOCA-B), modified Rankin Scale (mRS), and Patient Health Questionnaire-9 (PHQ-9) to assess post-stroke cognition, functional recovery, and depression, respectively. Data were entered and analyzed using SPSS software version 25. A binary logistic regression model was employed to identify the predictors of post-stroke cognitive impairment. A p-value of 0.05 was considered statistically significant.

**Results:**

Of the 79 stroke survivors approached, 67 were included. The mean (SD) age was 52.1 (12.7) years. Over half (59.7%) of the survivors were males and most (67.2%) were urban residents. The median stroke duration was 3 years ranging from 1 to 4 years. Almost half (41.8%) of stroke survivors had cognitive impairment. Increased age (AOR=0.24, 95% CI=0.07,0.83), lower education (AOR=4.02, 95% CI=1.13,14.32), and poor function recovery (mRS ^3^3) (AOR=0.27, 95% CI=0.08-0.81) were predictors significantly associated with post-stroke cognitive impairment.

**Conclusion:**

Nearly one in two stroke survivors had cognitive impairment. The major predictors associated with cognitive decline were age above 45 years, low literacy, and poor recovery in physical function. Although causality cannot be inferred, physical rehabilitation and better education are essential in building cognitive resilience among stroke survivors.

## Introduction

Stroke remains one of the major causes of mortalities and most common causes of neurological disabilities among the elderlies worldwide. According to the 2019 Global Burden of Disease study, the age-standardized stroke related mortality and morbidity risks were 3.6 and 3.7 times higher in low-income groups than in high-income groups, respectively (1). Other than this, stroke survivors often suffering from profound cognitive dysfunction and neuropsychiatric changes with adverse outcomes on patients functionality and quality of life (2). Studies have reported stroke increases the risk of cognitive impairment 5 to 8 times (3) when compared with the general population including the lifetime risk of developing either stroke, dementia or both is approximated 1 in 3 persons worldwide (4). Post-stroke cognitive impairment (PSCI) is a multidomain disruption of cognitive function, including executive function, attention, concentration, language, memory, and visuospatial ability, associated with stroke (5). The severity of PSCI exists in a continuum from post-stroke mild cognitive impairment (PS-MCI) to post-stroke dementia (PSD).

Some studies have documented that the prevalence of PSCI to vary based on different settings, study designs, sample size, patient’s characteristics, diagnostic criteria, and assessment methods (6). In the western countries, the prevalence of cognitive impairment 3-months after stroke ranges from 22% in England (7) to 47.3% in France (8). Recent studies in Africa reported PSCI of 20% in Benin at 6 months after stroke (9) and 50% in Ghanaian stroke survivors after 2 years (10).

There ae several factors influencing PSCI. Those factors are ranging from demographic (age, gender, educational attainment), pre-stroke physical and cognitive status, index stroke features including hemorrhagic and recurrent strokes, post-stroke complications such as infection, delirium, depression and early seizures, and neuroimaging predictors such as cerebral small-vessel disease, cortical atrophy and medial temporal lobe atrophy. All these factors may all conspire to differentially influence the trajectory of cognitive impairment after stroke (11, 12).

The issue of stroke and other non-communicable diseases (NCDs) such as dementia are increasing due to the rapid epidemiological transition, lifestyle changes and population aging in most sub-Saharan Africa (SSA) countries (11). Despite the increment of the problem, there is a dearth of information regarding the epidemiology and burden of stroke related cognitive impairment. With this background information, we assessed the magnitude and predictors of post-stroke cognitive impairment in Ethiopian stroke survivors attending the outpatient clinics in three neurology referral clinics at the capital, Addis Ababa, Ethiopia to generate evidence that would contribute for the control and prevention strategies in the country.

## Methods

### Study design, area, and period

A facility based cross-sectional study was conducted at the outpatient neurology clinics of three neurology referral centers namely Tikur Anbessa Specialized Hospital (TASH), Lancet General Hospital (LGH) and Yehuleshet Specialty Clinic (YSC) between February and June 2021. TASH was established in 1974 and it is the largest teaching and referral hospital in Ethiopia that serves as the only neurology training center in the country. LGH is a multi-specialty private hospital and YSC is a well-established private neurology center in the capital, Addis Ababa and provides a comprehensive neurology service by qualified neurologists to all patients referred to the center from elsewhere.

### Study participants

The main eligibility criteria for enrollment of patients were adult stroke survivors (18 years and above) who had a confirmed diagnosis of stroke and came for follow-up at least three months post-stroke and agreed to participate in the study. Patients with severe aphasia, known diagnosis of severe psychiatric illness or dementia, prior hearing and visual deficits and refused to participate were excluded.

### Sample size and sample technique

The sample size was determined using G-power software. A power analysis for a one-tailed chi-test indicated that the minimum sample size to yield a statistical power of at least 0.95 with an alpha of 0.05 and a medium effect size of 0.4 (because we do not know the appropriate effect size) is 82. Patients fulfilling the above criteria were enrolled in order of arrival until the required sample size is attained.

### Data collection tool and procedure

The important data were collected using a pretested structured interviewer-administered questionnaire containing socio-demographic, cardiovascular risk factors, and clinical features of stroke from patients and/or their caregivers. The cognitive test tools were prepared in English and forward translated into the local language (Amharic) and back translated to English to maintain its consistency. The Amharic version was pretested on 5% of the study participants to check the clarity of the questions and amended as appropriate.

Before data collection, five neurology residents who served as data collectors were trained for two days in the objective of the study and interviewing techniques to standardize their method of data collection. The collected data were checked every day for completeness and consistency by the investigators.

#### Cognitive assessment

Cognitive evaluation was done using the short form of informant questionnaire for Cognitive Decline in the Elderly (short IQCODE) to assess the pre-stroke cognitive status though in the final analysis it was dropped as two-third of the participants presented without an informant.

**Post-stroke cognitive status** (mild cognitive impairment (MCI) or dementia) was evaluated using the Montreal Cognitive Assessment – Basic (MOCA-B), which has been validated for screening MCI in low literacy (< 6 years of education) and uneducated individuals in several languages (13). The tool is a 30-point test and evaluates six cognitive domains (visual perception, executive functioning, language, attention, memory, and orientation). Literacy based MOCA-B cutoff classified patients with scores 17–22 for literate (14–19 for illiterate or under 6-year education) to have MCI, and ≤ 16 (≤ 13 for illiterate or under 6-year education) to have dementia, while scores of ≥ 23 for literate and ≥ 20 for illiterate and low education level were indicative of normal cognitive performance. (14).

**Functional status** was evaluated by the modified Rankin Scale (mRS), a six-point ordinal scale ranging from 0 (no symptoms) to 6 (death) and measures the degree of disability or dependence in everyday life, including instrumental and basic activities of daily living. It was categorized into good (mRS < 3) and poor (mRS ≥3) functional recovery.

**Post-stroke depression** was assessed using the Patient Health Questionnaire (PHQ-9) employed to screen for post stroke depression. The 9-item scale was scored as no depression = 0, mild depression = 1–9, moderated depression = 10–14 and severe depression = 15–27.

### Data analysis

Data were processed and analyzed using the Statistical Package for Social Sciences (SPSS) software Version 25 (IBM-SPSS Inc., Chicago, IL, USA). Descriptive statistics: mean, median, standard deviation (SD), range, frequency and proportion were calculated. Student t-test and Chi-square or Fisher’s Exact test were used for comparing continuous and categorical variables, respectively. Independent predictors of post-stroke cognitive decline were assessed by univariate and multivariate logistic regression model and results were presented using odds ratio (OR), 95% confidence interval (CI) and p value. Statistically significant was considered with p value < 0.05.

## Results

### Baseline characteristics of stroke participants

A total of 79 stroke survivors were approached, 12 were excluded due to severe aphasia and established hearing deficits, thus the final analysis was based on 67 patients. The mean (SD) age was 52.1 (12.7) years ranging from 23 to 77 years. There were slightly more males (59.7) than females (40.3%). About two-thirds (65.7%) were married and living with either their families - spouse and/or children (80.6%). More than half of the participants had either secondary (22.4%) or college education (35.8%), while 16.4% had only a primary education. The rest 25.4% were illiterate with no formal education. Majority (67.2%) of the participant were from an urban setting and 29.8% had an office job. Around a third of the participants had a monthly income level under the poverty line (57 USD per month). (Table 1).

### Vascular risk factors and PSCI in stroke survivors

As shown in Table 2, hypertension (30%) was the major vascular risk factor identified followed by dyslipidemia (8.9%) and diabetes (7.4%). Infectious risks such as HIV and latent syphilis were identified in 8.7% and 4.4%, respectively. More than half (58.2%) of the participants had hospital admission and 17.9% had stroke related medical complications such as seizure and aspiration pneumonia. Although majority of the patients had first-ever stroke, 19.4% had a recurrence. Post stroke cognitive impairment was identified in 41.8% of stroke survivors and a third (30%) of them had MCI while the rest 12% had post stroke dementia.

**Figure 1:**
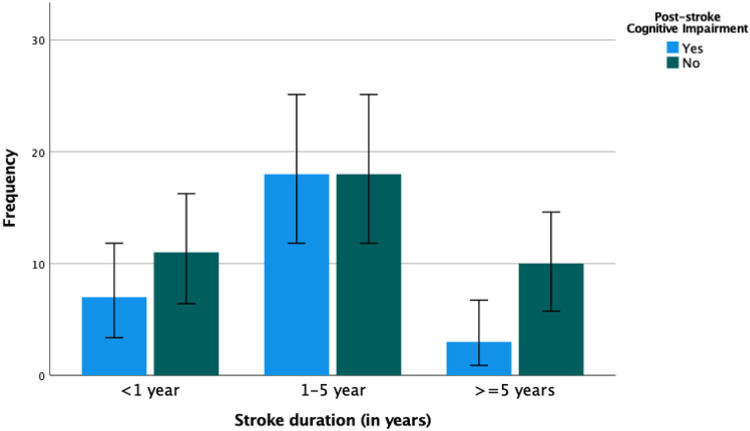
Cluster bar graph showing decreasing frequency of cognitive impairment with increased duration after stroke

Although statistically significance was not achieved, when patients were stratified by the disease duration the magnitude of cognitive dysfunction grow exponentially over the first five years and later it dropped. (Fig. 1)

The median duration [IQR] since index stroke was 36 [12–50] months which ranges from 4 to 132 months. Thirty-nine patients were admitted to the hospital after stroke episode and of those, 38.5% developed PSCI. As shown in Table 3 there were significant differences in the average age (SD) and average mRS score (SD) of patients who developed PSCI as compared to those without PSCI.

As depicted in Table 4, both in bivariate and multivariate logistic regression analysis, older age, low or lack of education, and poor functional recovery were significantly associated with PSCI. Stroke survivors with low or lack of education had 4 times increased risk for cognitive impairment when compared with educated patients (AOR = 4.02, 95% CI = 1.13,14.32). In addition, being middle and old aged (≥ 45 years) (AOR = 0.24, 95% CI = 0.07,0.83) and having poor recovery on motor functions (mRS ≥3) (AOR = 0.27, 95% CI = 0.08–0.81) were significantly associated with PSCI. However, the association between post-stroke cognitive dysfunction and gender, living arrangement, address and stroke subtypes did not reach the significance level.

**Figure 2:**
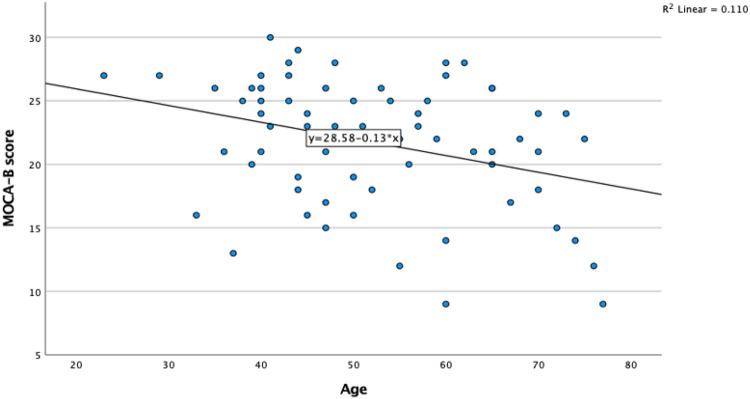
Scatter Plot showing the MOCA-B score decreases with increasing age

Figure 2 showed the statistically significant inverse correlation between increasing age and lower MOCA-B mean score (r=−0.33, R^2^ = 0.11, p = 0.006) (Fig. 2).

## Discussion

In the present study, 42% of stoke survivors experienced some form of cognitive impairment which included PS-MCI in 30% and PSD in 12% with about in 3.4 years of average duration after the stroke incident. Our findings are comparable with prior studies conducted in high income countries which reported 32% in United Kingdom (15), 39% in Australia (16) and 47.3% in France (8) as well as in low and middle income countries such as Chile (39%) (17), Ghana (7.6%) (10) and Nigeria (48.3%) (18). On the other hand, compared with other HICs our findings are lower than Portugal (42 vs 55%) (19), Norway (42 vs 57%) (20) and South Korea (42 vs 69.8%) (21). Such differences might be attributed to the study settings and the sample used.

A community-based study by Qu Y et al showed the highest post stroke cognitive dysfunction at 80.9% where 49% had PSCI without dementia and 32% with PSD (22). This variation can be ascribed to the differences in the study design (institution-based), study area (urban setting), study participants (one third had stroke in the young), stroke duration (median of 3 years), and assessment method (MoCA-B) used in our study. Generally, the magnitude of PSCI reported in our study is similar to that in the existing literature.

Although stroke risk increases with age, the average age of stroke survivors in our cohort was 52 years and more than one-third had stroke at a younger age, which is defined as stroke occurrence before the age of 45 years. This result is in agreement with our prior study in the same setting (23). However, most other regional and global studies reported a relatively higher age for stroke related cognitive impairment (9, 10, 18, 20). This might be explained by the Ethiopia population demography where more than two-third of the population is young. which is predominantly male, and males often developed stroke at earlier age as compared to females. Similar to other African studies (10, 18), majority (60%) of our study participants were males. This could be attributed to the reality that in low-income countries such as Ethiopia males are the bread winners and financial decision makers of the house and this gives them the upper hand to seek better health services as compared to the females.

Several sociodemographic and clinical variables contribute to the cognitive outcome of stroke survivors. Among these, increasing in age was documented as the major predictor of PSCI in prior studies (2, 12, 18). Similarly to the aforementioned studies, we have observe a significant association between stroke onset at the age of 45 years and above and PSCI though our cohort is relatively younger. Likewise, a Ghanaian study identified that the risk of PSCI increased by 44% for every 10-year increase in age and the authors attribute this to possible synergistic interaction between the vascular insult and neurodegenerative process which ends up in cognitive dysfunction (10). In the present study almost half of the participants had little or no education and this was independently associated with PSCI with 4 times higher risk as compared to the literate group. A case-control study from Nigeria (18) reported that lower education was associated with 5 times increased risk of PSCI. To the contrary, better education increases the tenacity of brain function by improving cognitive reserve and this serves as a surrogate marker for cognitive reserve.

It is known that functional recovery is a proxy marker for stroke severity and clinical deficit, stroke location and vascular burden, and acute stroke care and rehabilitation (10, 12). Interestingly, we found a significant association between poor function recovery and PSCI. A meta-analysis of randomized controlled trials (24) revealed that a structured physical activity and neurorehabilitation training enhances cognitive performance after stroke as early as in 12 weeks. Therefore, one way to address the growing burden of stroke on cognitive and physical outcomes of patients is by incorporating a comprehensive and multimodal rehabilitation interventions at an early stage of stroke.

## Strength and limitations of the study

Firstly, this study is the first of its kind to assess the magnitude of cognitive impairment in a sample of stroke survivors from Ethiopia. Secondly, we had attempted to show the association between cognitive dysfunction and demographic, clinical and stroke related factors. Nevertheless, the study had some limitations that include use of small sample size, the nature of the design employed and the study settings which were limited to three urban neurology centers which might have over represented the severe cases and therefore needs to be interpreted cautiously. The pre-stroke cognitive evaluation by short IQCODE was not considered in the analysis due to incomplete data.

## Conclusion

Our study found a relatively higher magnitude of cognitive dysfunction among males than female stroke survivors in Ethiopia. The major predictors identified were age 45 years and above, low educational attainment and poor recovery of physical function. Although causality cannot be inferred, physical rehabilitation and better education might play a role in building cognitive resilience among stroke survivors.

## Figures and Tables

**Table 1:** Socio-demographic characteristics of stroke survivors in Ethiopia (n = 67)

Variable	Frequency (%)
**Age in years (mean, SD)**	52.1 (± 12.7)
**Age group**	25 (37.3)
≤ 45 years	
> 45 years	42 (62.7)
**Gender**	
Male	40 (59.7)
Female	27 (40.3)
**Marital status**	44 (65.6)
Married	
Single	9 (13.4)
Widowed	7 (10.5)
Divorced	7 (10.5)
**Living arrangement**	54 (80.6)
With families	
Alone	9 (13.4)
With relatives	3 (4.5)
Nursing home	1 (1.5)
**Educational attainment**	17 (25.4)
No formal education	
Primary education	11 (16.4)
Secondary education	15 (22.4)
College and above	24 (35.8)
**Occupation**	20 (29.9)
Employed (Government office)	
Retired	11 (16.4)
Housewife	10 (14.9)
Merchant	9 (13.4)
Daily laborer	11 (16.4)
Unemployed	4 (6)
Farmer	2 (3)
**Monthly average household income**	25 (37.3)
<2000 ETB (< 57 USD)	
>2000 ETB (> 57 USD)	42 (62.7)
**Living address**	45 (67.2)
Urban	
Rural	22 (32.8)

* ETB: Ethiopian Birr; USD: United States Dollar

**Table 2:** Stroke risk factors and clinical profiles of stroke survivors in Ethiopia (N = 67)

Variable	Post-stroke Cognitive Impairment		Total (%)
	Yes (%)	No (%)	
	28 (41.8%)	39 (58.2%)	
Hypertension	20 (30)	25 (37.3)	45 (67.2)
Diabetes mellitus	5 (7.4)	6 (8.9)	11 (16.4)
Dyslipidemia	6 (8.9)	11 (16.4)	17 (25.4)
Smoking	4 (5.9)	11 (16.4)	15 (22.4)
Alcohol misuse	4 (5.9)	5 (7.5)	9 (13.4)
Obesity	4 (5.9)	4 (5.9)	8 (11.9)
HIV infection	4 (5.9)	2 (2.9)	6 (8.9)
Latent syphilis	2 (2.9)	1 (1.5)	3 (4.4)
Hospital admission	15 (22.4)	24 (35.8)	39 (58.2)
Post stroke medical complication	4 (5.9)	8 (11.9)	12 (17.9)
Recurrent stroke	7 (10.4)	6 (8.9)	13 (19.4)

**Table 3:** Mean age and mean score difference in some variables between PSCI and those without PSCI

Variables	PSCI		Total	P value
	Yes	No		
Age ± SD	56.6 ± 12.9	48.9 ± 11.6	52.1 ± 12.6	0.013*
Stroke duration (months)	32.9 ± 26.6	43.5 ± 36.41	39.14 ± 32.9	0.20
Duration of hospitalization (days)	14.6 ± 17.3	17.9 ± 13.8	16.6 ± 15.2	0.51
mRS score	2.3 ± 1.2	1.7 ± 1.1	1.9 ± 1.2	0.04*
PHQ-9 score	1.7 ± 1.1	1.4 ± 1.1	1.5 ± 1.1	0.19

* Statistically significant

**Table 4:** Univariate and multivariate logistic regression of the determinant variables for PSCI (N = 67)

Variables	Bivariate		Multivariate	
	COR (95% CI)	P value	AOR (95% CI)	P value
Gender				
Male	1		1	
Female	0.83 (0.31–2.23)	0.72	0.74 (0.26–2.16)	0.58
Age group				
< 45 years	1		1	
≥ 45 years	0.28 (0.09–0.86)	0.02	0.24 (0.07–0.83)	0.02*
Literacy				
Literate	1		1	
Low education- illiterate	5.1 (1.53–16.94)	0.005	4.02 (1.13–14.32)	0.03*
Living arrangement				
With family	1		1	
Alone	0.66 (0.15–2.9)	0.58	0.57 (0.12–2.87)	0.5
Income				
>57 USD	1		1	
<57 USD	1.5 (0.55–4.08)	0.43	1.43 (0.49–4.14)	0.51
Home address				
Urban	1		1	
Rural	0.9 (0.29–2.79)	0.85	0.71 (0.21–2.43)	0.58
Stroke subtype				
Hemorrhagic	1		1	
Ischemic	0.69 (0.22–2.17)	0.53	1.95 (0.55–6.83)	0.29
Functional recovery (mRS)				
Good (mRS < 3)	1		1	
Poor (mRS ≥3)	3.33 (1.16–9.53)	0.02	0.27(0.08–0.81)	0.02*
Post stroke depression				
No-mild	1			
Moderate-severe	1.95 (0.71–5.33)	0.19	0.61 (0.19–1.95)	0.4

* Statistically significant, USD: United States dollar, mRS; modified Rankin Scale

## Abbreviations

ETB: Ethiopian Birr
IQCODE: informant questionnaire for Cognitive Decline in the Elderly
LGH: Lancet general hospital
MCI: Mild cognitive impairment
MOCA-B: Montreal cognitive assessment-Basic
mRS: modified Rankin scale
PHQ-9: Patient health questionnaire-9
PSCI: Post-stroke cognitive impairment
PSD: Post-stroke dementia
TASH: Tikur Anbessa Specialized hospital
USD: United States dollar
YSC: Yehuleshet specialty clinic
